# Optimization in continuous phase‐transition extraction of crude flavonoids from finger citron fruit and evaluation on their antiaging activities

**DOI:** 10.1002/fsn3.1450

**Published:** 2020-02-18

**Authors:** Haiqiang Chen, Jing Wang, Xiaojuan Liu, Aimei Zhou, Jie Xiao, Kaixin Huang, Hanmin Chen, Yong Cao

**Affiliations:** ^1^ Guangdong Provincial Key Laboratory of Nutraceuticals and Functional Foods College of Food Science South China Agricultural University Guangzhou China; ^2^ Guangdong Zhancui Food co., Ltd. Chaozhou China

**Keywords:** antiaging activities, *Caenorhabditis elegans*, continuous phase‐transition extraction, finger citron fruit, flavonoids

## Abstract

The development of antiaging functional products is a hot topic in the field of functional foods. However, the efficient extraction of functional ingredients is the limiting step for the functional food industry. Continuous phase‐transition extraction (CPE) is a new extraction technique that combines the advantages of Soxhlet extraction and supercritical extraction, which may have a distinct advantage over traditional methods in the extraction of flavonoids. In our study, the Box–Behnken design combined with response surface methodology was used to optimize CPE of crude flavonoids from finger citron fruit. The antiaging activities of finger citron crude flavonoids (FCCF) were evaluated by *Caenorhabditis elegans* (*C. elegans*) model. The optimal extraction conditions for CPE were as follows: ethanol concentration 85%, temperature 90°C, time 120 min, and pressure 0.2 MPa. Compared with the heat reflux extraction, the extraction rate and content of FCCF extracted by CPE increased by 24.28% and 33.22% (*p* < .05), respectively. FCCF extended the lifespan of *C. elegans* by 14.94% without causing adverse effects on their reproduction and locomotion ability. A further analysis suggested that FCCF prolonged the lifespan of nematodes under normal and oxidative stress by increasing the activity of major enzymes in endogenous antioxidant defense system and reducing the accumulation of reactive oxygen species (ROS) and malondialdehyde (MDA). The results confirmed the effectiveness of CPE in extracting crude flavonoids from finger citron fruit, and the extracted FCCF exhibited strong antiaging activities.

## Graphical abstract

Compared with heat reflux extraction, continuous phase‐transition extraction can significantly increase the extraction rate and content of finger citron crude flavonoids (FCCF). FCCF may prolong the lifespan of *Caenorhabditis elegans* under normal and oxidative stress by increasing the activity of major enzymes in endogenous antioxidant defense system and reduce the accumulation of reactive oxygen species (ROS) and malondialdehyde (MDA).

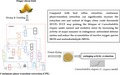

**Highlights**

- Continuous phase‐transition extraction (CPE) was used to extract flavonoids.
- Compared with heat reflux extraction (HRE), CPE can significantly increase the extraction rate and content of crude flavonoids.
- Finger citron crude flavonoids (FCCF) have excellent anti‐aging activities.
- FCCF can increase the activity of superoxide dismutase (SOD) and catalase (CAT) of *Caenorhabditis elegans* and reduce the accumulation of reactive oxygen species (ROS) and malondialdehyde (MDA).

## 1 INTRODUCTION

Aging is an extremely complex process, and the cellular manifestation theory of aging proposes that reactive oxygen species (ROS) produced in cells can cause aging (Marchi et al., 2012). However, ROS are involved in the modification of various cellular responses and by‐products of aerobic respiration. In addition, under nutrient deficiency, inflammation, ultraviolet (UV), and exposure to heavy metals and oxidants, cells produce ROS. Excessive production of ROS leads to significant apoptosis or necrosis of cells (Kim et al., 2016). During cell differentiation, ROS and free radicals affect the expression and signal transduction pathways of various genes (Pacurari et al., 2008). Cells use antioxidants (such as vitamin C, vitamin E, and carotenoids) and antioxidant defense systems including superoxide dismutase (SOD), catalase (CAT), glutathione reductase (GR), and glutathione peroxidase (GPx) to combat the effects of ROS and free radicals (MatÉs, Pérez‐Gómez, & Castro, 1999). Recently, many studies have reported that extracts of plant resources (e.g., fruits and vegetables) can effectively eliminate excessive ROS and free radicals (Gao et al., 2019; Gedikoğlu, Sökmen, & Çivit, 2019; Wei, Zhan, Chen, Xie, & Fang, 2019), and the development of natural antiaging functional foods derived from plants has thus attracted growing research interest.

Finger citron fruit [*Citrus medica* L. var.* sarcodactylis* (Noot.) Swingle], Rutaceae family, is a citron variety that looks like an “open hand” when their fruit is ripe. In China and Japan, finger citron fruit has been regarded as an adjuvant herbal medicine to treat a variety of diseases for decades (Peng et al., 2009). Nowadays, due to its rich flavonoids, finger citron fruit has attracted intensive attention in the scientific community for the treatment of nonalcoholic fatty liver, aging, inflammatory, and schizophrenia (Lascala et al., 2018; Pozzo et al., 2018). Flavonoids, important secondary metabolites of plants, are the most common polyphenols in the human diet, which protect multiple organs from oxidation in the body and are considered as natural antioxidants (Granato et al., 2018; Prasain, Carlson, & Wyss, 2010). Antioxidants have been proven to effectively reduce the damaging effects rooting from ROS on the body and delay many events that cause cellular aging (Nemzer et al., 2014). Notably, flavonoid extraction process determines whether finger citron fruit can be developed into a high‐quality functional food. However, traditional flavonoid extraction methods (such as heat reflux extraction, Soxhlet extraction, and hot water extraction) are limited by drawbacks including time consuming, costly, low recovery, toxic reagent residues, and even loss of flavonoids (Tang et al., 2017; Zhang, Zheng, & Zhang, 2019). Therefore, it is urgent to develop an environmentally friendly and effective method for extracting flavonoids from finger citron fruit.

**Figure 1:**
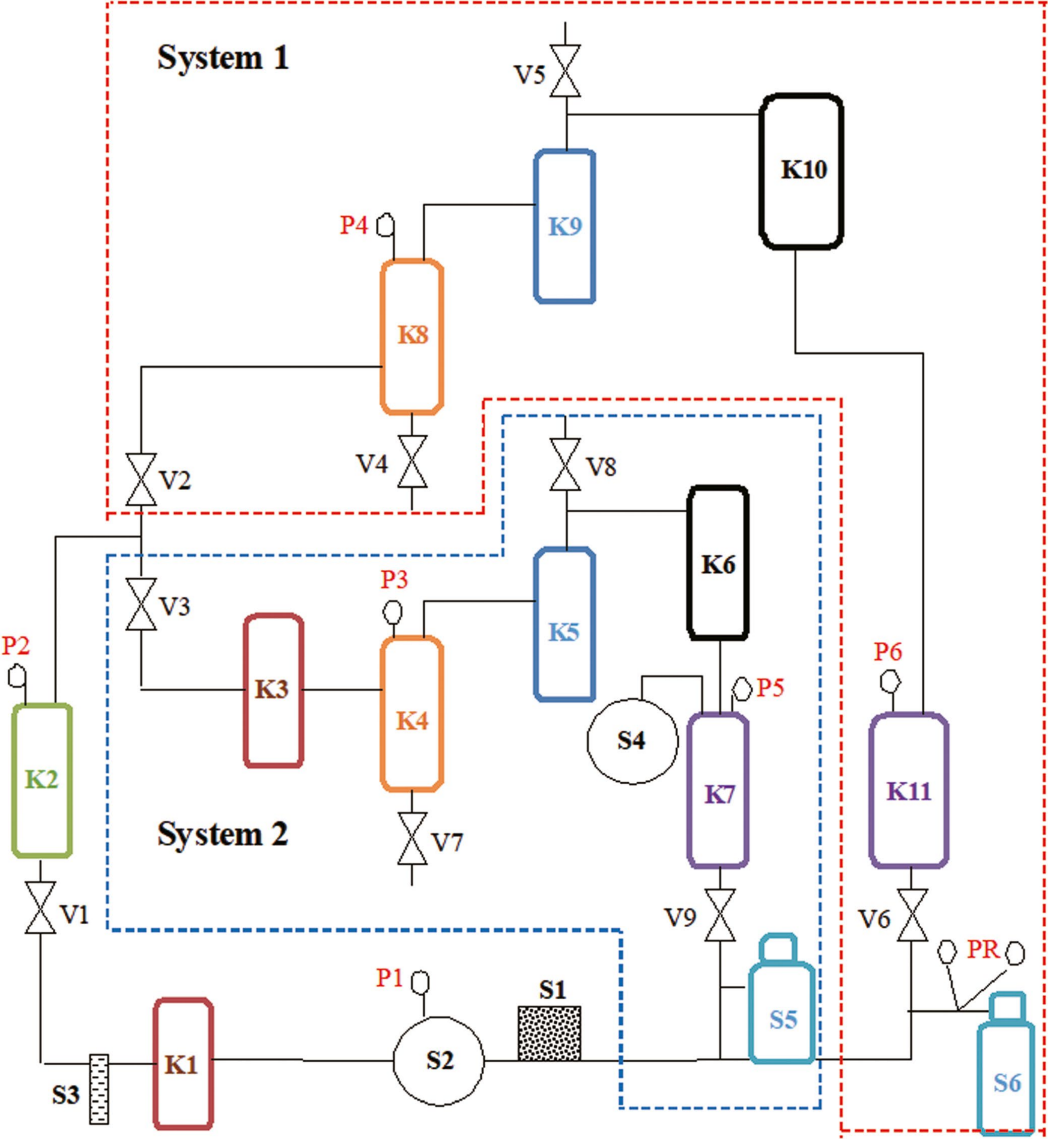
Schematic diagram of continuous phase‐transition extraction system. S1: microfilter; S2: high pressure pump; S3: flowmeter; S4: vacuum pump; S5 and S6: ethanol; V*i* (*i* = 1, 2, 3 to 9): valve; P*i* (*i* = 1, 2, 3 to 6): pressure indicator; K1 and K3: heat exchanger; K2: extraction pot; K4 and K8: separation pot; K5 and K9: purification column; K6 and K10: condenser; K7 and K11: solvent storage pot

Continuous phase‐transition extraction (CPE) developed by our team is a new type of extraction apparatus for functional ingredients (Figure 1). The CPE apparatus includes a desorption system and a solvent recovery system, in which the extraction solvent undergoes continuous phase change, recycling, and purification in a closed and low‐pressure system (Miao et al., 2013). The gaseous extractant was first compressed into liquid by high pressure pump and heater, which extracted target components through an extraction vessel containing sample. The solvent extractant containing the extract was then pumped into a vacuum evaporation and separation tank for conversion to gas and separation from the extract. Finally, the extractant was re‐condensed into a pure solvent and the cycle was repeated until the extraction was complete. CPE combines the advantages of Soxhlet extraction and supercritical extraction. Its extraction solvent not only has higher dissolving power than traditional extraction methods, but also does not cause saturation problems. The extraction process of CPE is performed at a relatively low temperature, and the extraction solvent can be recovered. These advantages may lead to superiority over traditional methods in extracting flavonoids.

*Caenorhabditis elegans* (*C. elegans*) is one of the most popular models for evaluating antiaging activities owing to its relatively short lifespan, ease of cultivation, and high homology to mammalian especially human biochemical and genetic pathways (Kaletta & Hengartner, 2006). *Caenorhabditis elegans* has been extensively used in studying the antiaging activities of natural active products such as *Opuntia* fruit extracts (Alejandra, Samanta, Francisco, & Fernando, 2019), black tea (Yuan et al., 2018), and grape pomace extracts (Ayuda‐Durán et al., 2019). In this study, CPE was used to extract crude flavonoids from finger citron fruit compared with the heat reflux extraction (HRE). The extraction process was optimized using Box–Behnken design (BBD) and response surface methodology (RSM) to achieve maximum crude flavonoid yield. The extraction yield and content of the crude finger citron crude flavonoids (FCCF) were analyzed. Furthermore, the antiaging activity was evaluated using *C. elegans* model.

## 2 MATERIALS AND METHODS

### 2.1 Materials and reagents

Dry finger citron fruit slices were provided by Zhancui Food Co. Ltd. The samples were further dried using hot air at 40°C for 24 hr to ensure the moisture content was <15%. The dried samples were then ground into powder with a particle size of 30 mesh by grinding machine (DLF30; Wenzhou Dingli Medical Instrument Co. Ltd), which was immediately packed in the vacuum polyethylene bag and stored in refrigerator at −20°C for further use.

Hesperidin, 2,7‐dichlorodihydrofluorescein diacetate (H2DCF‐DA) and paraquat were purchased from Sigma Chemical Co. Experimental *C. elegans*: wild‐type N2 (var. Bristol) was obtained from the Caenorhabditis Genetics Center (CGC; University of Minnesota, Minneapolis, Minn., USA). The uracil mutant *Escherichia coli* OP_50_ (*E. coli* OP_50_) was provided by the College of Resource and Environment, South China Agricultural University. All other chemicals used were of analytical grade.

### 2.2 Experimental design

A schematic diagram of the CPE experimental setup was shown in Figure 1.

Based on the results of single‐factor experiments (date not shown), the experimental design for optimizing CPE process for FCCF was carried out using BBD and RSM. Since extraction pressure showed the highest extraction yield at 0.2 MPa and had no further effect on the extraction yield above 0.2 MPa in single‐factor experiments, the other three independent variables (concentration of alcohol, temperature, and time) were selected for optimization. For a BBD with three independent variables at three levels, 17 experimental runs were required as presented in Table 1. The following second‐order polynomial model was used to fit the experimental data:

(1) $$Y=\kappa_{0}+\sum_{i=1}^{3}\kappa_{i}x_{i}+\sum_{i=1}^{3}k_{\mathrm{ii}}x_{i}^{2}+\sum \sum_{i<j}\kappa_{\mathrm{ij}}x_{i}x_{j}$$

where *Y* was the estimated response; *x_i_* and *x_j_* were independent variables; and $\kappa_{0},\kappa_{i},\kappa_{\mathrm{ii}}$, and *k_ij_* were regression coefficients of intercept, linear, quadratic, and interaction terms, respectively.

**Table 1:** Experimental conditions for BBD and the corresponding responses measured

RUNS	Ethanol concentrations (*X*_1_, %)	Temperature (*X*_2_, °C)	Time (*X*_3_, min)	FCCF extraction yield (%)
1	0 (75)	1 (90)	1 (150)	1.57 ± 0.01
2	0 (75)	1 (90)	−1 (90)	1.55 ± 0.10
3	−1 (65)	−1 (60)	0 (120)	1.07 ± 0.03
4	0 (75)	0 (75)	0 (120)	1.52 ± 0.02
5	1 (85)	1 (90)	0 (120)	1.68 ± 0.00
6	−1 (65)	0 (75)	1 (150)	1.15 ± 0.02
7	0 (75)	0 (75)	0 (120)	1.51 ± 0.01
8	0 (75)	0 (75)	0 (120)	1.51 ± 0.00
9	−1 (65)	0 (75)	−1 (90)	1.06 ± 0.02
10	0 (75)	−1 (60)	−1 (90)	0.54 ± 0.02
11	−1 (65)	1 (90)	0 (120)	1.00 ± 0.11
12	0 (75)	0 (75)	0 (120)	1.49 ± 0.02
13	0 (75)	0 (75)	0 (120)	1.46 ± 0.03
14	1 (85)	0 (75)	−1 (90)	0.92 ± 0.03
15	0 (75)	−1 (60)	1 (150)	1.14 ± 0.04
16	1 (85)	−1 (60)	0 (120)	0.45 ± 0.02
17	1 (85)	0 (75)	1 (150)	1.42 ± 0.06

Abbreviation: FCCP, finger citron crude flavonoids.

Heat reflux extraction was performed as a control experiment. According to the preliminary optimized investigation (data not shown), the optimum HRE condition was as follows: temperature 80°C, time 90 min, ethanol concentration 73%, and liquid to solid ratio 31:1.

### 2.3 Analysis of extraction yield and content of FCCF

The extraction yield of FCCF was determined according to the method of Qin and Chen (2018) with some modifications. Briefly, 1 ml extraction sample liquid was diluted to 5 ml with a blank solution reagent (citric acid–NaOH buffer solution with pH 6.0) and then mixed thoroughly with 5 ml of 90% (*w*/*v*) diethylene glycol solution and 0.1 ml of 160 g/L NaOH solution in 10‐ml test tube with glass stopper. The mixture was placed in a water bath at 40°C for 10 min and cooled in a cold‐water bath for 5 min. The absorbance was determined at 420 nm against a reagent blank prepared similarly without NaOH solution by a UV–vis spectrophotometer (UV‐3010; HITACHI). The yield of FCCF was calculated according to the standard curve equation of hesperidin (*A* = 0.006*C*−0.0002, the correlation coefficient *r* was .9999). Furthermore, the extraction sample liquid was lyophilized to calculate the content of FCCF. The extraction yield and content of FCCF in the sample liquid were calculated according to the following equation:

(2) $$\text{Extraction}\;\text{yields}\;\text{of}\;\text{FCCF}=\frac{\rho \times v}{m\times f}\times 100\%$$

(3) $$\text{Crude}\;\text{flavonoids}\;\text{content}=\frac{\rho \times v}{m_{1}\times f}\times 100\%$$

where *ρ* (mg/ml) was the hesperidin concentration calculated according to the standard curve; *v* (ml) was the total volume of extraction sample liquid;was dilution factor of the sample solution; *m* (g) was the dry weight of the finger citron fruit powder during the extraction; and *m*_1_ (g) was the weight of the extract after freeze‐drying.

### 2.4 Determination of antiaging activity of FCCF

#### 2.4.1 *Caenorhabditis elegans* culture and synchronization

*Caenorhabditis elegans* culture was carried out as the method described by Onken and Driscoll (2010). A nematode growth medium (NGM) plate having *E. coli* OP_50_ was propagated at 20°C for *C. elegans* and synchronized with sodium hypochlorite according to the reported literature (Rathor, Pant, Awasthi, Mani, & Pandey, 2017).

#### 2.4.2 Preparation of treatment plates

*Escherichia coli* OP50 strain was inoculated on LB broth medium (2.1 g of LB broth in 100 ml of distilled water) and then cultured at 37°C with shaking at 170 rpm for 12 hr (to OD_600_ = 0.4) to obtain *E. coli* OP50 solution. 5 μl of CPE‐derived FCCF at 200 μg/ml (in distilled water, *w*/*v*) and H_2_O (control) were filtered to remove bacteria, mixed with 95 μl *E. coli* OP_50_ solution, and then inoculated onto NGM (65 mm × 10 mm) plates to feed worms.

#### 2.4.3 Lifespan assay

Lifespan measurements of *C. elegans* were performed at 20°C on NGM plates (with *E. coli* OP_50_) in a temperature‐controlled incubator according to the method of Liu, Luo, et al. (2016). Synchronized L4 larvae (at least 100 wild worms per treatment) were transferred to fresh NGM plates with 5 μl of FCCF from CPE at concentration of 200 μg/ml (the optimal dose on lifespan extension of *C. elegans*, date not shown) or H_2_O (control). Lifespan was calculated from the first day of L4 larvae. After the fourth day, the worms were transferred to a new plate for 2 days to ensure adequate food and the number of dead worms was recorded (the criterion for worm death was no movement and swallowing, and remained unresponsive after touching). The median life is the sum of the lifespan of all worms divided by the total.

#### 2.4.4 Reproduction assay

Reproduction assay was performed according to the previously reported method (Chen et al., 2014). Briefly, wild‐type *C. elegans* started from L4 larvae (10 individuals and two worms per plate) were transferred to fresh plates every 24 hr until the worms no longer laid eggs. The plates were placed at 20°C for 24 hr to incubate the eggs, and the number of worms was counted before the offspring hatched and grew up.

#### 2.4.5 Locomotion assay

The method of cultivating *C. elegans* in locomotion assay was similar to that of lifespan assays. Ten worms were randomly selected for motility determination on days 5, 10, and 15. The motility classification was scored by gently prodding the locomotion phenotype of *C. elegans* with platinum wire. Its behavior was classified as “A” (youthful, symmetrical, spontaneous movement), “B” (less smooth, uncoordinated movement, must be prodded frequently), or “C” (sway only head or tail if prodded) as described by Wu et al (Wu, Huang, & Xiang, 2019). Meanwhile, the sinusoidal movement of the worms was also analyzed by scoring the number of worm movements in a sinusoidal manner within 1 min on days 5, 10, and 15 (each plate selected seven worms for recording), while the head swing frequency was measured within 30 s on the 2nd and 6th days after transferring the L4 stage worms to each set of medium.

#### 2.4.6 ROS detection assay

Intracellular ROS levels were determined by the method of Shukla et al. (2012) with minor modifications. *Caenorhabditis elegans* was cultivated from eggs as described in lifespan assays. After 96 hr of treatment with 5 μl of 200 μg/ml CPE‐derived FCCF or H_2_O (control), the worms were placed on NGM plates three times to remove bacteria. The worms were then transferred to 96‐well fluorescent plates containing 50 μl of M9 buffer with 80 worms per well. Meanwhile, 50 μl fresh 100 μM H_2_DCF‐DA solution in M9 buffer was added to the well. On each plate, the well containing 50 μl of H_2_DCF‐DA solution and 50 μl of M9 buffer without worms was used as blank control. Fluorescence spectral measurements were captured by an EnSpire^®^ Multimode Plate Reader (PerkinElmer) at an excitation wavelength of 485 nm and an emission wavelength of 528 nm. Observations were recorded at 25°C for 6 hr at intervals of 20 min, and the results were expressed in relative fluorescence units (RFU).

#### 2.4.7 SOD, CAT, and MDA detection assay

The detection assay of SOD, CAT, and MDA was performed as the method described by Liu, Chen, Liu, and Cao (2018) with slight modifications. After treatment with 5 μl of FCCF from CPE for 96 hr or not (control), 200 worms were selected and washed three times in sterile water. After low‐speed centrifugation, the supernatant was discarded and the precipitate was added with 0.5 ml sterile water followed by homogenization ultrasonically at low temperature (repeated twice and 2 min each time at intervals of 1s for 1s homogenization) using a JY92‐2D Ultrasonic Cell Disruption System (Scientz). After sonication, 0.5 ml of 1% CHAPS solution (*w*/*v*) was added and centrifuged at 12,000 *g* for 15min at 4°C. The supernatant was collected and stored at 4°C until use. SOD, CAT, and MDA were measured using the SOD Assay kit (WST‐1 method), CAT Assay kit (Ultraviolet), and MDA Assay kit (Colorimetric method; Nanjing Institute of Bioengineering Institute, China), respectively, according to the provided protocols. Enzyme activities were expressed in units per mg of protein.

#### 2.4.8 Stress resistance assays

Heat shock and oxidative stress measurements were performed according to the method of Hansen, Hsu, Dillin, and Kenyon (2005) with a minor modification. The cultivation method of *Caenorhabditis elegans* was the same as the lifespan assays. After 96‐hr treatment on plates containing 5 μl of 200 μg/ml CPE‐resulted FCCF, the worms were transferred to 37°C for heat shock experiments. The number of worm deaths was recorded every 4 hr to plot the life curve. For oxidative stress assays, worms were transferred to plates containing 25 mg/ml (*w*/*v*) paraquat and the survival rate of the worms was recorded every 24 hr until all worms died. The standard of worm death was the same as that described in section 2.4.3. After oxidative stress treatment, ROS and MDA accumulation and the activity of SOD and CAT were determined in the same methods as those in 2.4.6 and 2.4.7.

### 2.5 Statistical analysis

All experiments were performed in triplicate, and the results were expressed as mean (±*SD*). BBD and RSM were established using the Design Expert 8.0.6 test software program (Stat‐Ease, Inc.), and the results of the experimental data were analyzed. Statistically significant differences (*p* < .05) in the experimental data were calculated by one‐way analysis of variance (ANOVA).

## 3 RESULTS AND DISCUSSION

### 3.1 Optimization of the CPE procedure for FCCF

According to the BBD design combined with RSM, 17 run experiments were conducted for the optimization of the extraction of FCCF by CPE and the results were shown in Table 1. The following equation was derived through multiple regression analysis to express the extraction yield of FCCF:

$$\begin{array}{cc} & Y=0.15+0.024X_{1}+0.33X_{2}+0.15X_{3}+0.32X_{1}X_{2}\\ & +0.10X_{1}X_{3}-0.14X_{2}X_{3}-0.26X_{1}^{2}-0.19X_{2}^{2}-0.11X_{3}^{2} \end{array}$$

where was the extraction yield of FCCF (%), was the ethanol concentrations (%), was the extraction temperature (°C), and was the extraction time (min).

Table 2 presented the results of variance (ANOVA) for the response surface quadratic model. In the experimental design, the *p*‐value of the model (*p* < .0001) was significant and the *p*‐value of lack of fit model (*p* = .1242) was not significant, suggesting that the model was suitable for prediction of the variation (Liu, Lou, et al., 2016). Meanwhile, the interaction of factors *X*_1_ *X*_2_ and the second‐order coefficient $X_{1}^{2}$, $X_{2}^{2}$ were highly significant as their *p*‐values were <.0001. The factors *X*_1_ *X*_3_, *X*_2_ *X*_3_ and the second‐order coefficient $X_{3}^{2}$ were significant as their *p*‐values were <.05. According the *p*‐values of the coefficients in the regression model, the order of the independent variables affecting the extraction yield of FCCF was ethanol concentrations (*X*_1_)> temperature (*X*_2_)> time (*X*_3_). The coefficient of determination (*R* ^2^) was used to determine whether the response surface model was workable. The value of *R* ^2^ was .9940, while the adjusted determination coefficient (Adj. *R* ^2^) value was .9862, indicating that the prediction model was in good agreement with the extraction yield of FCCF during experiment.

**Table 2:** ANOVA statistics of the quadratic model for the extraction yield of FCCF

Source	Coefficient estimate	Sum of squares	Degree of freedom	Mean square	*F*‐value	*p*‐Value
Model	1.50	2.11	9	0.23	127.96	<.0001
*X*_1_‐ethanol concentrations	0.024	4.51	1	4.51	2.47	.1603
*X*_2_‐extraction temperature	0.33	0.85	1	0.85	461.93	<.0001
*X*_3_‐extraction time	0.15	0.18	1	0.18	100.05	<.0001
*X*_1_ *X*_2_	0.32	0.42	1	0.42	230.96	<.0001
*X*_1_ *X*_3_	0.10	0.042	1	0.042	22.97	.0020
*X*_2_ *X*_3_	−0.14	0.084	1	0.084	45.97	.0003
$X_{1}^{2}$	−0.26	0.27	1	0.27	149.96	<.0001
$X_{2}^{2}$	−0.19	0.16	1	0.16	85.52	<.0001
$X_{3}^{2}$	−0.11	0.047	1	0.047	25.50	.0015
Residual		0.013	7	1.83		
Lack of fit		0.011	3	3.508	3.59	.1242
Pure error		2.280	4	5.700		
Cor total		2.12	16			

Note

The coefficients are given in coded units. *R* ^2^ = .9940, $R_{\text{adj}}^{2}$ = .9862.

FCCP, finger citron crude flavonoids.

**Figure 2:**
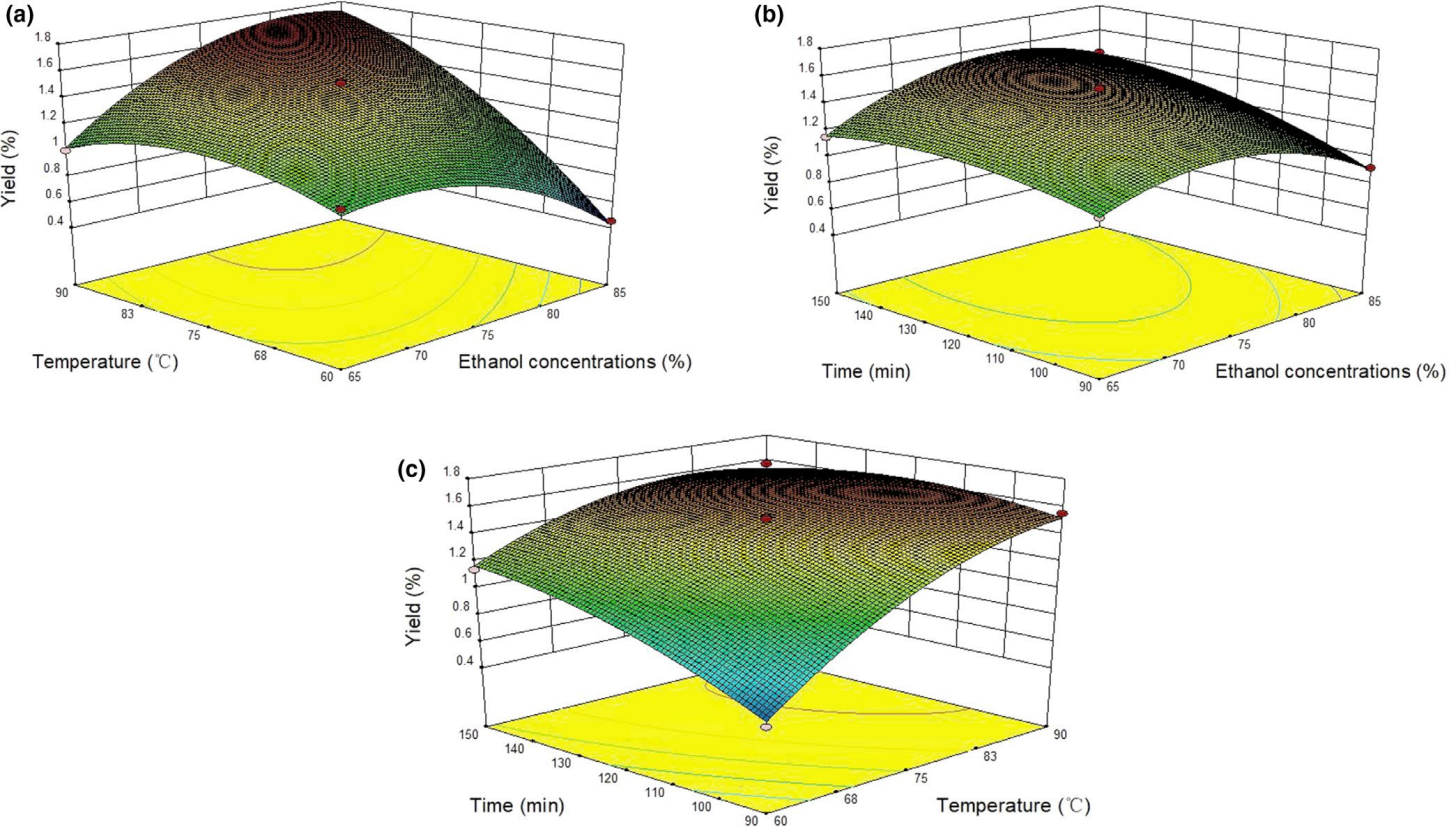
Results of response surface analysis: effect of ethanol concentrations, extraction temperature, and time on yield. (a) Temperature and ethanol concentrations at 0.2 MPa, 120 min; (b) time and ethanol concentrations at 0.2 MPa, 80°C; and (c) time and temperature at 0.2 MPa, ethanol concentration 75%

The 3D response surface plots (Figure 2) were generated using Design Expert software to study the interaction of ethanol concentration, extraction temperature, and time on the extraction yield of FCCF. The extraction yields first increased and then decreased with the increase in ethanol concentrations. When the concentration of ethanol exceeded 75%, the extraction yields slowly decreased. Possible reason might be that 75% aqueous ethanol provided the most suitable polarity for extraction of FCCF. In addition, the swelling of plant materials by water increased with the contact surface area between the plant matrix and the solvent (Zhao, Zhang, Li, Dong, & Liu, 2015). The extraction yield of FCCF increased with the increase in temperature. Temperature was a very influential factor in the extraction yield of FCCF. However, the extraction yield of FCCF did not further increase when the extraction temperature was higher than 80°C. With the increase in extraction temperature, the diffusion rates and solubility of the extraction solvents increased (Liu et al., 2011). Moreover, heat treatment may soften plant tissues and weaken the interaction between flavonoid–protein and flavonoid–polysaccharides; therefore, more flavonoids would migrate to solvent (Mokrani & Madani, 2016). This explained the increase in extraction yield of FCCF when increasing temperature. Extraction time showed a similar effect on FCCF extraction yield to that of temperature. The long‐time extraction made the sample and solvent well mixed hence increased the extraction yield of FCCF, but the extraction yield did not further increase when the extraction time exceeded 120 min.

According to the BBD model equation, the optimum parameters of the CPE process for FCCF were as follows: the ethanol concentration of 85%, the extraction temperature of 90°C, the extraction time of 120 min, and the extraction pressure of 0.2 MPa. Under these conditions, the extraction yield of FCCF was 1.69%, which had no significant difference from the predicted value (1.72%; *p* > .05), indicating that the mathematical model established by the experiment was reliable.

### 3.2 Extraction yield and content of FCCF

**Figure 3:**
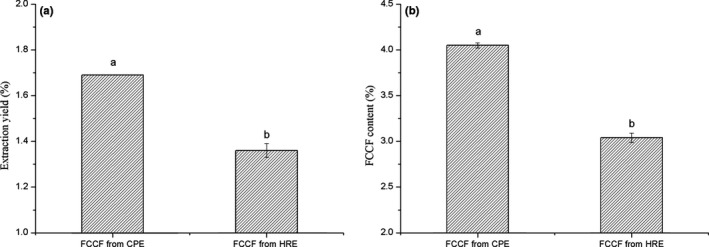
Extraction yield (a) and content (b) of finger citron crude flavonoids (FCCF) by CPE and HRE methods. Data represent mean ± *SD* of three independent experiments. Values with different letters are significantly different (*p* < .05)

In order to evaluate extraction efficiency of CPE, we extracted FCCF by HRE at the optimum extraction condition. According to Figure 3, the extraction yield and content of the FCCF in the CPE were significantly higher than that of the HRE (*p* < .05), which was increased by 24.28% and 33.22%, respectively. The higher efficiency could be attributed to the continuous extraction process, which ensured that the extraction solvent was unsaturated and the extraction was performed more sufficiently and efficiently (Peng et al., 2009). In addition, the entire process of CPE was carried out under airtight and low‐pressure conditions, and it was multifunctional, safe, and reliable. Therefore, CPE was a more effective method for FCCF extraction.

### 3.3 Antiaging activity of FCCF

#### 3.3.1 Effects of FCCF on the lifespan of *Caenorhabditis elegans*

**Figure 4:**
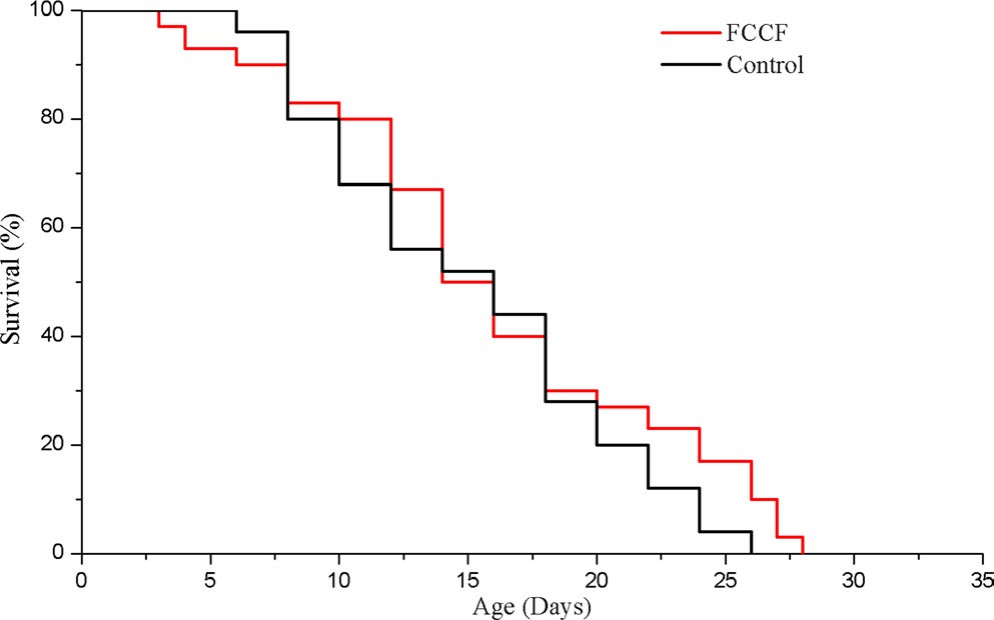
Effects of finger citron crude flavonoids (FCCF) at 200 μg/ml on the fraction survival of *Caenorhabditis elegans*. Survival curves of N2 wild‐type worms raised at 20°C on plates containing either FCCF or H2O (control). Day 0 refers to the first day *C. elegans* at L4 stage were transferred to NGM plates. Three independent trials were conducted

Lifespan was the main antiaging indicator of *C. elegans*. According to our previous experiments, 200 μg/ml FCCF from CPE was the optimal dose on lifespan extension of *C. elegans* (data are not shown), so 200 μg/ml FCCF was chosen for further experiment. As described in Figure 4, at a culture temperature of 20°C, FCCF was manifested with better survival curves and prolonged the median lifespan of worms by 14.94% as compared to the control, but there was no significant difference between the two groups (*p* > .05; Table 3). The lifespan for *C. elegans* is approximately 3–4 weeks, and it is considered as a significant difference if the lifespan of is extended for one or 2 days (Liu, Luo, et al., 2016).

**Table 3:** Lifespan analysis: effect of FCCF on median and maximum lifespan of wild type (N2)

Strains	Treatment	Median lifespan (Mean ± *SD*) (days)	Maximum lifespan (Mean ± *SD*) (days)	Mean fold increase ％	*p* Value	Sample size
N2 (20°C)	Control	15.66 ± 1.35	16.96 ± 0.85			75
	FCCF	18.00 ± 2.26	19.6 ± 2.26	14.94	.2300	90

FCCP, finger citron crude flavonoids.

#### 3.3.2 Effects of FCCF on physiological functions of *Caenorhabditis elegans*

**Figure 5:**
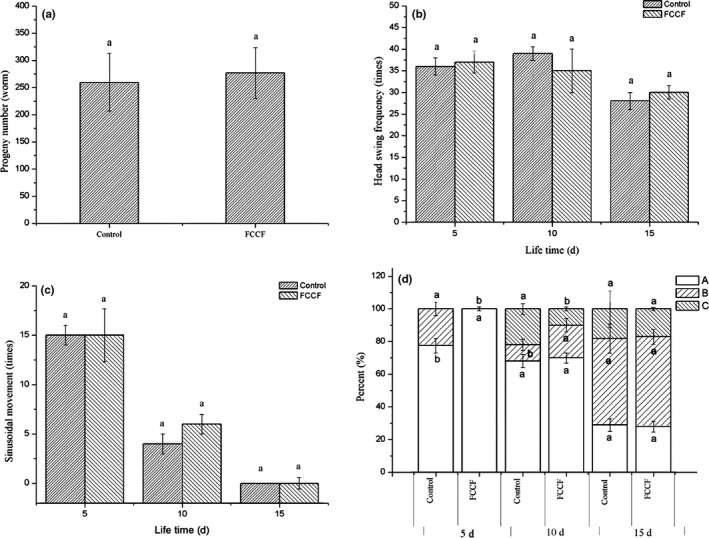
Effects of different treatments on physiological processes of *Caenorhabditis elegans*. (a) The progeny number of each worm was counted until the parental worms were dead or stopped producing progeny. The head swing frequency (b), sinusoidal movement (c), and body movement (d) were counted under a dissecting microscope for 30 s on the 5th, 10th, and 15th days of worms. In body movement, worm behavior was classified as “A” (youthful, symmetrical, spontaneous movement), “B” (less smooth, uncoordinated movement, must be prodded frequently), or “C” (sway only head or tail if prodded). During the same period, the different letters (a, b) of different treatment groups have significant differences (*p* < .05). Date represent means ± SD of three independent experiments (*N* = 3)

During the life process of *C. elegans*, their reproductive behavior and athletic ability are the most susceptible physiological responses to environmental factors (Kim, Jeon, & Cha, 2014) and the decline in physiological functions occurs in the aging process of *C. elegans* (Chen et al., 2014). To determine whether FCCF exerted adverse influences on the physiological functions of *C. elegans* at the optimal concentration, physiological function changes including the progeny production and locomotion ability of worms under FCCF treatment were analyzed (Figure 5). The results showed that the number of eggs laid per day by the worms in control group (259.50 ± 52.67) and FCCF‐treated group (276.75 ± 46.42) had no significant difference (*p* > .05; Figure 5a). When worms run out of their own sperm supply, the naturally occurring hermaphrodite terminates their reproduction process. These results indicated that FCCF prolonged the lifespan of *C. elegans* without sacrificing their ability to reproduce.

Along with the aging process, muscles of *C. elegans* gradually degenerate, weakening their ability of moving in solid and liquid media (Kim et al., 2014). To determine the relationship between worm's locomotion ability and lifespan, we measured the effect of FCCF on different movement characteristics at early (on days 5), middle (on days 10), and late life stages (on days 15). As shown in Figure 5b,c, the worm's head swing frequency and sinusoidal motion decreased with time, but the normal sinusoidal motion decreased more obviously than the head swing frequency. The worms almost lost the ability of sinusoidal motion in late life stage. However, there was no significant difference in head swing frequency and sinusoidal movement between the FCCF‐treated group and the control. Figure 5d depicted three types of movement in worms fed with or without FCCF (classification of A, B, and C; see methods). As the worms aged, the spontaneous (A) movement was found to gradually decrease, but FCCF treatment could significantly delay the decline of class A movement in the early life stage (*p* < .05). Class B movement occurred in the early life stage of the control group, while the FCCF‐treated group appeared in the middle life stage. There were three types of movements in the middle life stage of the worms, and the proportions of class A, B, and C movement in the control group were 68.11 ± 3.98%, 9.96 ± 3.50%, and 21.94 ± 3.35%, while in the FCCF‐treated group, their proportions were 70.00 ± 3.20%, 20.00 ± 4.20%, and 10.00 ± 1.20%, respectively, The proportion of class B and C movement is remarkably lower than that of the control (*p* < .05). In the late life stage, there was no significant difference (*p* > .05) in the proportion of the three classes of movements between the two groups, and class B movement was dominated in this stage. As the worms aged, their movements appeared to be sluggish, uncoordinated, and gradually decreased until they stopped moving and eventually died (Vayndorf, Lee, & Liu, 2013). These findings indicated that FCCF did not affect the ability of worm's movement and increased the locomotion ability to some extent.

#### 3.3.3 Effects of FCCF on ROS, MDA accumulation, and enzyme activity of *Caenorhabditis elegans*

**Figure 6:**
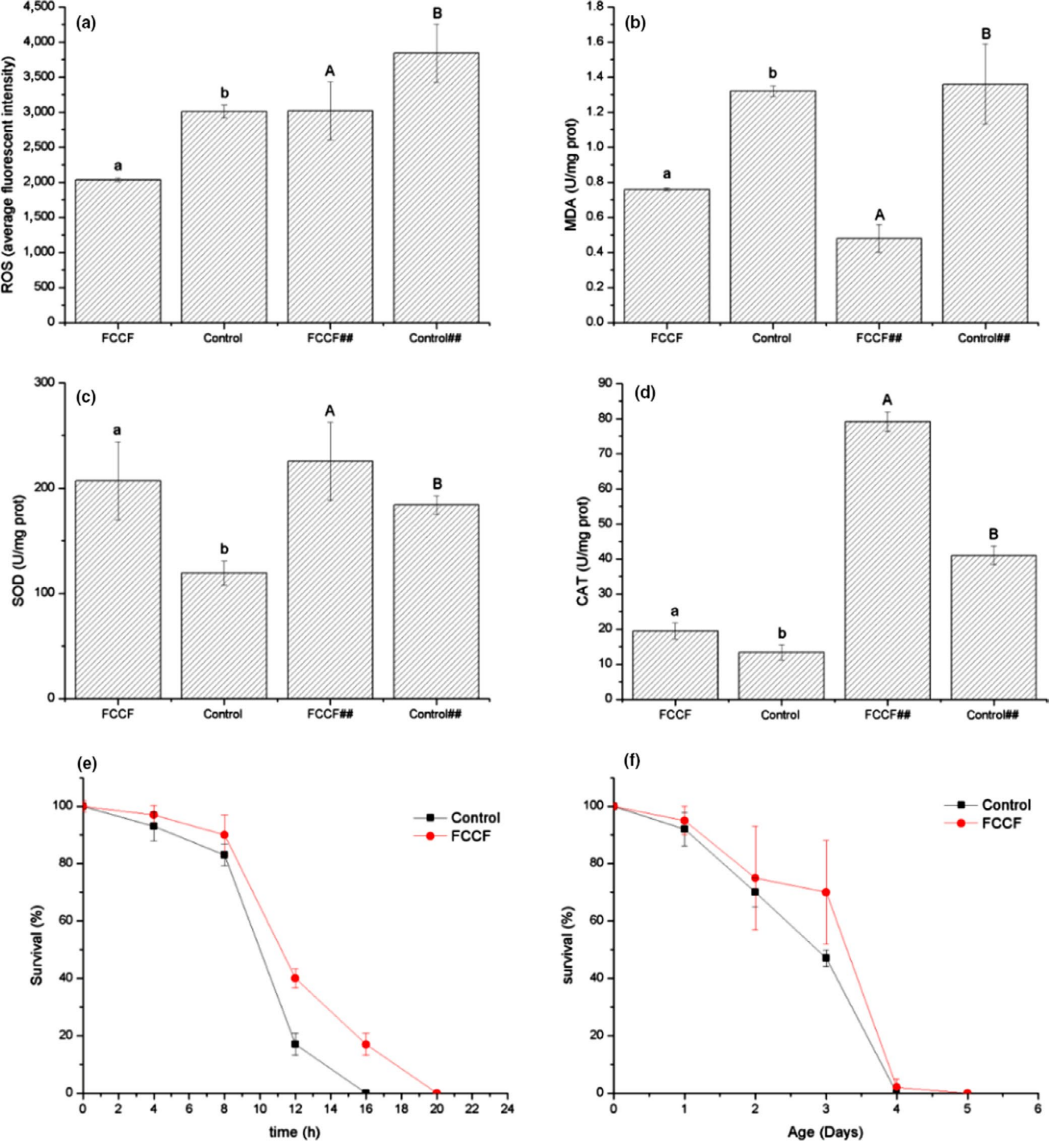
Effect of pretreatment with finger citron crude flavonoids (FCCF) on reactive oxygen species (ROS) and malondialdehyde (MDA) accumulation, enzyme activity, and resistance to stress in *Caenorhabditis elegans*. After 96 hr of treatment with FCCF or control, 200 worms were collected for sample preparation. ROS (a) and MDA (b) accumulation was measured according to the Materials and Methods 2.4.6 and 2.4.7. SOD (c) and CAT (d) activity was expressed as units/mg of protein. Values with different letters are significantly different (*p* < .05 for “a” comparisons “b” or “A” comparisons “B”). ##The *C. elegans* were exposed to 25 mM Paraquat. Worms were pretreated with FCCF and survived significantly longer after 37°C heat shock (e; *N* ≥ 30 animals per group) and exposure to 25 mg/ml paraquat (f; *N* ≥ 30 animals per group). Three independent trials were performed. Data are expressed as the mean ± *SD* of three independent experiments (*N* = 3)

The mitochondrial electron transport chain of worm's cell produces large amounts of ROS free radicals in the oxidative damage environment (Zhang et al., 2016), and the accumulation of ROS free radicals accelerates the aging of worm's tissues and organs (Wu et al., 2019). Malondialdehyde (MDA) is one of the end products of ROS‐induced lipid peroxidation and an important biomarker of oxidative stress. We used the net rate of intracellular ROS production by H_2_DCF‐DA to detect whether FCCF reduced ROS in *C. elegans*. As illustrated in Figure 6a,b, FCCF significantly reduced the accumulation of ROS in worms (*p* < .05), which was 37.44% lower than that of the control group. This suggested that FCCF have the ability to reduce ROS levels in worm's cell due to their potent antioxidant capacity. The scavenge effect of FCCF on MDA in worms was consistent with the trend of ROS. Compared with the control group, the MDA content in FCCF‐treated group decreased by 56.06% (*p *< .05), indicating that FCCF reduced lipid peroxidation. These results were in accordance with the prolonged lifespan of the worm's exposure to FCCF. Long‐term accumulation of ROS causes damage to worm cells, which leads to disease, disorders, and death (Sonane, Moin, & Satish, 2017). Reducing ROS levels in worms by FCCF may be a useful strategy to prevent or delay these pathological processes. Therefore, we deduced that lifespan prolongation of *C. elegans* might be partially related to the reduced accumulation of intracellular ROS and MDA rooting from FCCF treatment.

Superoxide dismutase and catalase in worms are the main enzymes in endogenous antioxidant defense system, which play an important role in protecting cells from oxidative damage by reactive oxygen species (Hunter et al., 2015). To investigate whether FCCF had a regulatory effect on endogenous antioxidant defense enzymes, we determined SOD and CAT activities in worms as shown in Figure 6c,d. The activities of SOD and CAT enzymes in the worms treated with FCCF were significantly higher than those in the control (*p* < .05), which were increased by 103.15% and 81.91%, respectively. These suggested that FCCF treatment improved the activity of SOD and CAT enzymes. Excessive ROS can cause serious damage to cellular molecules, which must be inactivated by different endogenous antioxidant enzymes, such as SOD, GPO, and CAT (Koch, Havermann, Büchter, & Wätjen, 2014). These results indicated that FCCF might contribute to the elimination of excess ROS and MDA by improving SOD and CAT enzyme activity, thus playing an antiaging role.

#### 3.3.4 Effects of FCCF on the stress resistance of *Caenorhabditis elegans*

Previous studies have demonstrated a correlation between the lifespan and antistress capabilities of *C. elegans* and other animals, including mammals (Gems & Partridge, 2008; Vayndorf et al., 2013). To determine whether FCCF have the ability to extend worm lifespan under environmental stress, *C. elegans* were challenged by stressors including heat shock and oxidative stress. The survival of the worms treated by FCCF increased compared with the control worms under heat shock, especially after 12 hr, at which the survival was significantly difference between the two groups (*p* < .05; Figure 6e). In addition, it was observed that the survival rate of worms treated with FCCF increased by 25% under heat shock conditions, indicating that FCCF conferred thermal stress resistance to worms. This result was consistent with other studies in *C. elegans*, which showed that plant‐derived extracts enhanced thermotolerance and prolonged lifespan (Benedetti et al., 2008).

After exposure to 25 mg/ml paraquat, the survival rate of worms also increased, and the survival rate increased by 25% compared to the control group (Figure 6f). The results showed that FCCF treatment improved the worms' resistance to oxidative stress. Under the oxidative stress, FCCF exhibited a protective effect against paraquat‐treated worms.

In order to determine how FCCF enhanced the stress resistance of *C. elegans* under oxidative stress, the effect of FCCF on radical scavenging capacity and enzyme activity in worms was evaluated. Paraquat was an intracellular ROS generator, so we conducted paraquat‐induced oxidative stress on *C. elegans* (Akhoon, Pandey, Tiwari, & Pandey, 2016). The observations showed that the total ROS level in the worms treated with FCCF was significantly reduced by 21.41% compared with the control group (*p* < .05; Figure 6a). Moreover, FCCF significantly reduced the content of MDA (*p* < .05) in worms, which was reduced by 64.71%, indicating that FCCF reduced the lipid peroxidation of worms under oxidative stress induced by paraquat. On the other hand, FCCF significantly increased the SOD and CAT enzyme activities in worms by 22.58% and 93.05% (*p* < .05), respectively (Figure 6c,d). These results were consistent with the findings of Wang et al. (2016). These results demonstrated that FCCF improved the antioxidant defense system of worms by modulating antioxidant enzyme activity and lipid peroxidation. Furthermore, the results suggested that FCCF extended the lifespan by enhancing the stress resistance of *C. elegans* under oxidative stress, and FCCF might obtain this effect by reducing the accumulation of intracellular ROS and MDA and increasing the activity of SOD and CAT enzymes.

## 4 CONCLUSIONS

In the present study, the process of extracting FCCF by CPE was optimized, and the antiaging activities were evaluated. Response surface methodology successfully optimized the extraction process. Compared with the HRE method, the CPE extracts had a significant increase in extraction yield and content of FCCF. The lifespan for *C. elegans* exposed to FCCF was improved without causing obvious, adverse effects on physiological functions including reproduction and locomotion ability. When exposed to different environmental stresses (oxidation and thermal stress), FCCF treatment extended lifespan by enhancing the stress resistance of *C. elegans*. A further analysis showed that FCCF prolonged the lifespan of nematodes under normal and oxidative stress by increasing the activity of major enzymes in endogenous antioxidant defense system, such as superoxide dismutase (SOD) and catalase (CAT), and reducing the accumulation of reactive oxygen species (ROS) and malondialdehyde (MDA). These results showed that CPE was a promising technique process for extracting flavonoids from finger citron fruit. CPE‐extracted FCCF showed strong antiaging activities and can be developed to natural antiaging functional foods.

## CONFLICT OF INTEREST

The authors declare that they do not have any conflict of interest.

## ETHICAL APPROVAL

This study does not involve any human or animal testing.

## References

[fsn31450-bib-0001] Akhoon, B. A. , Pandey, S. , Tiwari, S. , & Pandey, R. (2016). Withanolide A offers neuroprotection, ameliorates stress resistance and prolongs the life expectancy of *Caenorhabditis elegans* . Experimental Gerontology, 78, 47–56. 10.1016/j.exger.2016.03.004 26956478

[fsn31450-bib-0002] Alejandra, G. R. M. , Samanta, H. G. , Francisco, G. C. , & Fernando, G. H. (2019). Extension of life‐span using a RNAi model and in *vivo* antioxidant effect of *Opuntia* fruit extracts and pure betalains in *Caenorhabditis elegans* . Food Chemistry, 274, 840–847. 10.1016/j.foodchem.2018.09.067 30373018

[fsn31450-bib-0003] Ayuda‐Durán, B. , González‐Manzano, S. , Gil‐Sánchez, I. , Moreno‐Arribas, M. V. , Bartolomé, B. , Sanz‐Buenhombre, M. , … González‐Paramás, A. M. (2019). Antioxidant characterization and biological effects of grape pomace extracts supplementation in *Caenorhabditis elegans* . Foods, 8, 75 10.3390/foods8020075 PMC6406641 30781355

[fsn31450-bib-0004] Benedetti, M. G. , Foster, A. L. , Vantipalli, M. C. , White, M. P. , Sampayo, J. N. , Gill, M. S. , … Lithgow, G. J. (2008). Compounds that confer thermal stress resistance and extended lifespan. Experimental Gerontology, 43, 882–891. 10.1016/j.exger.2008.08.049 18755260 PMC2603168

[fsn31450-bib-0005] Chen, Y. , Onken, B. , Chen, H. , Xiao, S. , Liu, X. , Driscoll, M. , … Huang, Q. (2014). Mechanism of longevity extension of *Caenorhabditis elegans* induced by pentagalloyl glucose isolated from eucalyptus leaves. Journal of Agricultural and Food Chemistry, 62, 3422–3431. 10.1021/jf500210p 24701969

[fsn31450-bib-0006] Gao, C. , Wang, F. , Yuan, L. , Liu, J. , Sun, D. , & Li, X. (2019). Physicochemical property, antioxidant activity, andcytoprotective effect of the germinated soybean proteins. Food Science & Nutrition, 7, 120–131. 10.1002/fsn3.822 30680165 PMC6341154

[fsn31450-bib-0007] Gedikoğlu, A. , Sökmen, M. , & Çivit, A. (2019). Evaluation of *Thymus vulgaris* and *Thymbra spicata* essential oils and plant extracts for chemical composition, antioxidant, and antimicrobial properties. Food Science & Nutrition, 7, 1704–1714. 10.1002/fsn3.1007 31139383 PMC6526640

[fsn31450-bib-0008] Gems, D. , & Partridge, L. (2008). Stress‐response hormesis and aging: “That which does not kill us makes us Stronger”. Cell Metabolism, 7, 200–203. 10.1016/j.cmet.2008.01.001 18316025

[fsn31450-bib-0009] Granato, D. , Shahidi, F. , Wrolstad, R. , Kilmartin, P. , Melton, L. D. , Hidalgo, F. J. , … Finglas, P. (2018). Antioxidant activity, total phenolics and flavonoids contents: Should we ban in vitro screening methods? Food Chemistry, 264, 471–475. 10.1016/j.foodchem.2018.04.012 29853403

[fsn31450-bib-0010] Hansen, M. , Hsu, A. , Dillin, A. , & Kenyon, C. (2005). New genes tied to endocrine, metabolic, and dietary regulation of lifespan from a *Caenorhabditis elegans* genomic RNAi screen. Plos Genetics, 1, e17 10.1371/journal.pgen.0010017 PMC1183531 16103914

[fsn31450-bib-0011] Hunter, G. J. , Trinh, C. H. , Bonetta, R. , Stewart, E. E. , Cabelli, D. E. , & Hunter, T. (2015). The structure of the *Caenorhabditis elegans* manganese superoxide dismutase MnSOD‐3‐azide complex. Protein Science, 11, 1777–1788. 10.1002/pro.2768 PMC4622211 26257399

[fsn31450-bib-0012] Kaletta, T. , & Hengartner, M. O. (2006). Finding function in novel targets: *C. elegans* as a model organism. Nature Review Drug Discovery, 5, 387–399. 10.1038/nrd2031 16672925

[fsn31450-bib-0013] Kim, D. K. , Jeon, H. , & Cha, D. S. (2014). 4‐Hydroxybenzoic acidmediated lifespan extension in *Caenorhabditis elegans* . Journal of Functional Foods, 7, 630–640. 10.1016/j.jff.2013.12.022

[fsn31450-bib-0014] Kim, D. , Shin, G. , Kim, J. , Kim, Y. , Lee, J. , Lee, J. S. , … Lee, O. (2016). Antioxidant and anti‐ageing activities of citrus‐based juice mixture. Food Chemistry, 194, 920–927. 10.1016/j.foodchem.2015.08.094 26471635

[fsn31450-bib-0015] Koch, K. , Havermann, S. , Büchter, C. , & Wätjen, W. (2014). *Caenorhabditis elegans* as model system in pharmacology and toxicology: Effects of flavonoids on redox‐sensitive signalling pathways and ageing. The Scientific World Journal, 2014, 920398 10.1155/2014/920398 24895670 PMC4032668

[fsn31450-bib-0016] Lascala, A. , Martino, C. , Parafati, M. , Salerno, R. , Oliverio, M. , Pellegrino, D. , … Janda, E. (2018). Analysis of proautophagic activities of Citrus flavonoids in liver cells reveals the superiority of a natural polyphenol mixture over pure flavones. Journal of Nutritional Biochemistry, 58, 119–130. 10.1016/j.jnutbio.2018.04.005 29890411

[fsn31450-bib-0017] Liu, H. , Zhang, Y. , Li, Q. , Zou, Y. , Shao, J. , & Lan, S. (2011). Quantification of lutein and zeaxanthin in margigold (*Tagetes erecta L*.) and poultry feed by ultra‐performance liquid chromatography and high performance liquid chromatography. Journal of Liquid Chromatography Related Technologies, 34, 2653–2663. 10.1080/10826076.2011.593220

[fsn31450-bib-0018] Liu, X. , Chen, X. , Liu, H. , & Cao, Y. (2018). Antioxidation and anti‐aging activities of astaxanthin geometrical isomers and molecular mechanism involved in *Caenorhabditis elegans* . Journal of Functional Foods, 44, 127–136. 10.1016/j.jff.2018.03.004

[fsn31450-bib-0019] Liu, X. , Lou, Q. , Cao, Y. , Goulette, T. , Liu, X. , & Xiao, H. (2016). Mechanism of different stereoisomeric astaxanthin in resistance to oxidative stress in *Caenorhabditis elegans* . Journal of Food Science, 81, 2280–2287. 10.1111/1750-3841.13417 27527357

[fsn31450-bib-0020] Liu, X. , Luo, Q. , Rakariyatham, K. , Cao, Y. , Goulette, T. , Liu, X. , & Xiao, H. (2016). Antioxidation and anti‐ageing activities of different stereoisomeric astaxanthin in vitro and in vivo. Journal of Functional Foods, 25, 50–61. 10.1016/j.jff.2016.05.009

[fsn31450-bib-0021] Marchi, S. , Giorgi, C. , Suski, J. M. , Agnoletto, C. , Bononi, A. , Bonora, M. , … Pinton, P. (2012). Mitochondria‐Ros crosstalk in the control of cell death and aging. Journal of Signal Transduction, 2012(329635), 17 10.1155/2012/329635 PMC3235816 22175013

[fsn31450-bib-0022] MatÉs, J. M. , Pérez‐Gómez, C. , & Castro, I. N. D. (1999). Antioxidant enzymes and human diseases. Clinical Biochemistry, 32, 595–603. 10.1016/S0009-9120(99)00075-2 10638941

[fsn31450-bib-0023] Miao, J. , Che, K. , Xi, R. , He, L. , Chen, X. , Guan, X. , … Cao, Y. (2013). Characterization and benzo[a]pyrene content analysis of camellia seed oil extracted by a novel subcritical fluid extraction. Journal of the American Oil Chemists Society, 90, 1503–1508. 10.1007/s11746-013-2293-1 24098057 PMC3785177

[fsn31450-bib-0024] Mokrani, A. , & Madani, K. (2016). Effect of solvent, time and temperature on the extraction of phenolic compounds and antioxidant capacity of peach (*Prunus persica L*.) fruit. Separation and Purification Technology, 162, 68–76. 10.1016/j.seppur.2016.01.043

[fsn31450-bib-0025] Nemzer, B. , Chang, T. , Xie, Z. , Pietrzkowski, Z. , Reyes, T. , & Ou, B. (2014). Decrease of free radical concentrations in humans following consumption of a high antioxidant capacity natural product. Food Science & Nutrition, 2, 647–654. 10.1002/fsn3.146 25493181 PMC4256568

[fsn31450-bib-0026] Onken, B. , & Driscoll, M. (2010). Metformin induces a dietary restriction‐like state and the oxidative stress response to extend *C. elegans* healthspan via AMPK, LKB1, and SKN‐1. PLoS One, 5, e8758 10.1371/journal.pone.0008758 20090912 PMC2807458

[fsn31450-bib-0027] Pacurari, M. , Yin, X. J. , Zhao, J. , Ding, M. , Leonard, S. S. , Schwegler‐Berry, D. , & Castranova, V. (2008). Raw single‐wall carbon nanotubes induce oxidative stress and activate MAPKs, AP‐1, NF‐kappaB, and Akt in normal and malignant human mesothelial cells. Environmental Health Perspectives, 116, 1211–1217. 10.1289/ehp.10924 18795165 PMC2535624

[fsn31450-bib-0028] Peng, C. , Ker, Y. , Weng, C. , Peng, C. , Huang, C. , Lin, L. , & Peng, R. Y. (2009). Insulin secretagogue bioactivity of finger citron fruit (*Citrus medica* L. var. *Sarcodactylis* Hort, Rutaceae). Journal of Agricultural and Food Chemistry, 57, 8812–8819. 10.1021/jf902143x 19761210

[fsn31450-bib-0029] Pozzo, E. D. , Leo, M. D. , Faraone, I. , Milella, L. , Cavallini, C. , Piragine, E. , … Martini, C. (2018). Antioxidant and antisenescence effects of bergamot juice. Oxidative Medicine and Cellular Longevity, 2018, 1–14. 10.1155/2018/9395804 PMC6079356 30116497

[fsn31450-bib-0030] Prasain, J. K. , Carlson, S. H. , & Wyss, J. M. (2010). Flavonoids and age‐related disease: Risk, benefits and critical windows. Maturitas, 66, 163–171. 10.1016/j.maturitas.2010.01.010 20181448 PMC2879453

[fsn31450-bib-0031] Qin, L. , & Chen, H. (2018). Enhancement of flavonoids extraction from fig leaf using steam explosion. Industrial Crops and Products, 69, 1–6. 10.1016/j.indcrop.2015.02.007

[fsn31450-bib-0032] Rathor, L. , Pant, A. , Awasthi, H. , Mani, D. , & Pandey, R. (2017). An antidiabetic polyherbal phytomedicine confers stress resistance and extends lifespan in *Caenorhabditis elegans* . Biogerontology, 18, 131–147. 10.1007/s10522-016-9668-2 27853905

[fsn31450-bib-0033] Shukla, V. , Yadav, D. , Phulara, S. C. , Gupta, M. M. , Saikia, S. K. , & Pandey, R. (2012). Longevity‐promoting effects of 4‐hydroxy‐E‐globularinin in *Caenorhabditis elegans* . Free Radical Biology and Medicine, 53, 1848–1856. 10.1016/j.freeradbiomed.2012.08.594 23000058

[fsn31450-bib-0034] Sonane, M. , Moin, N. , & Satish, A. (2017). The role of antioxidants in attenuation of *Caenorhabditis elegans* lethality on exposure to TiO_2_ and ZnO nanoparticles. Chemosphere, 187, 240–247. 10.1016/j.chemosphere.2017.08.080 28854380

[fsn31450-bib-0035] Tang, X. , Zhu, D. , Huai, W. , Zhang, W. , Fu, C. , Xie, X. , … Fan, H. (2017). Simultaneous extraction and separation of flavonoids and alkaloids from *Crotalaria sessiliflora* L. by microwave‐assisted cloud‐point extraction. Separation and Purification Technology, 175, 266–273. 10.1016/j.seppur.2016.11.038

[fsn31450-bib-0036] Vayndorf, E. M. , Lee, S. S. , & Liu, R. H. (2013). Whole apple extracts increase lifespan, healthspan and resistance to stress in *Caenorhabditis elegans* . Journal of Functional Foods, 5, 1235–1243. 10.1016/j.jff.2013.04.006 PMC3714114 23878618

[fsn31450-bib-0037] Wang, Q. , Huang, Y. , Qin, C. , Liang, M. , Mao, X. , Li, S. , … Huang, Z. (2016). Bioactive peptides from *Angelica sinensis* protein hydrolyzate delay senescence in *Caenorhabditis elegans* through antioxidant activities. Oxidative Medicine and Cellular Longevity, 2016, 8956981 10.1155/2016/8956981 26941890 PMC4752986

[fsn31450-bib-0038] Wei, Q. , Zhan, Y. , Chen, B. , Xie, B. , & Fang, T. (2019). Assessment of antioxidant and antidiabetic properties of *Agaricus blazei* Murill extracts. Food Science & Nutrition, 8, 1–8. 10.1002/fsn3.1310 PMC6977522 31993159

[fsn31450-bib-0039] Wu, W. , Huang, T. , & Xiang, F. (2019). Polyethylene glycol‐based ultrasonic‐assisted enzymatic extraction, characterization, and antioxidant activity in vitro and in vivo of polysaccharides from *Lonicerae japonica* leaves. Food Science & Nutrition, 7, 3452–3462. 10.1002/fsn3.1186 31741734 PMC6848850

[fsn31450-bib-0040] Yuan, P. , Pan, L. , Xiong, L. , Tong, J. , Li, J. , Huang, J. , … Liu, Z. (2018). Black tea increases hypertonic stress resistance in *C. elegans* . Food & Function, 9, 3798–3806. 10.1039/c7fo02017a 29932178

[fsn31450-bib-0041] Zhang, J. , Shi, R. , Li, H. , Xiang, Y. , Xiao, L. , Hu, M. , … Huang, Z. (2016). Antioxidant and neuroprotective effects of Dictyophora indusiata polysaccharide in *Caenorhabditis elegans* . Journal of Ethnopharmacology, 192, 413–422. 10.1016/j.jep.2016.09.031 27647012

[fsn31450-bib-0042] Zhang, L. , Zheng, D. , & Zhang, Q. (2019). Purification of total flavonoids from R*hizoma Smilacis Glabrae* through cyclodextrin‐assisted extraction and resin adsorption. Food Science & Nutrition, 7, 449–456. 10.1002/fsn3.809 30847122 PMC6392876

[fsn31450-bib-0043] Zhao, Z. , Zhang, Q. , Li, Y. , Dong, L. , & Liu, S. (2015). Optimization of ultrasound extraction of *Alisma orientalis* polysaccharides by response surface methodology and their antioxidant activities. Carbohydrate Polymers, 119, 101–109. 10.1016/j.carbpol.2014.11.052 25563949

